# Clinical application of plasma P-tau217 to assess eligibility for amyloid-lowering immunotherapy in memory clinic patients with early Alzheimer’s disease

**DOI:** 10.1186/s13195-024-01521-9

**Published:** 2024-07-06

**Authors:** Matthew D. Howe, Karysa J. Britton, Hannah E. Joyce, William Menard, Sheina Emrani, Zachary J. Kunicki, Melanie A. Faust, Brittany C. Dawson, Meghan C. Riddle, Edward D. Huey, Shorena Janelidze, Oskar Hansson, Stephen P. Salloway

**Affiliations:** 1 Butler Hospital Memory & Aging Program, 345 Blackstone Boulevard, Providence, RI 02906 USA; 2 Department of Psychiatry and Human Behavior, Brown University, Providence, RI USA; 3 Washington University in St. Louis, St. Louis, MO USA; 4 University of Pennsylvania Perelman School of Medicine, Philadelphia, PA USA; 5 Clinical Memory Research Unit, Clinical Sciences Malmö, Lund University, Lund, Sweden; 6 Memory Clinic, Skåne University Hospital, Malmö, Sweden

**Keywords:** Alzheimer’s disease, Blood biomarkers, Clinical research, Dementia, Immunotherapy

## Abstract

**Background:**

With the approval of disease-modifying treatments (DMTs) for early Alzheimer’s disease (AD), there is an increased need for efficient and non-invasive detection methods for cerebral amyloid-β (Aβ) pathology. Current methods, including positron emission tomography (PET) and cerebrospinal fluid (CSF) analysis, are costly and invasive methods that may limit access to new treatments. Plasma tau phosphorylated at threonine-217 (P-tau217) presents a promising alternative, yet optimal cutoffs for treatment eligibility with DMTs like aducanumab require further investigation. This study evaluates the efficacy of one- and two-cutoff strategies for determining DMT eligibility at the Butler Hospital Memory & Aging Program (MAP).

**Methods:**

In this retrospective, cross-sectional diagnostic cohort study, we first developed P-tau217 cutoffs using site-specific and BioFINDER-2 training data, which were then tested in potential DMT candidates from Butler MAP (total n = 150). ROC analysis was used to calculate the area under the curve (AUC) and accuracy of P-tau217 interpretation strategies, using Aβ-PET/CSF testing as the standard of truth.

**Results:**

Potential DMT candidates at Butler MAP (n = 50), primarily diagnosed with mild cognitive impairment (n = 29 [58%]) or mild dementia (21 [42%]), were predominantly Aβ-positive (38 [76%]), and half (25 [50%]) were subsequently treated with aducanumab. Elevated P-tau217 predicted cerebral Aβ positivity in potential DMT candidates (AUC = 0.97 [0.92–1]), with diagnostic accuracy ranging from 0.88 (0.76–0.95, *p* = 0.028) to 0.96 (0.86–1, *p* < .001). When using site-specific cutoffs, a subset of DMT candidates (10%) exhibited borderline P-tau217 (between 0.273 and 0.399 pg/mL) that would have potentially required confirmatory testing.

**Conclusions:**

This study, which included participants treated with aducanumab, confirms the utility of one- and two-cutoff strategies for interpreting plasma P-tau217 in assessing DMT eligibility. Using P-tau217 could potentially replace more invasive diagnostic methods, and all aducanumab-treated participants would have been deemed eligible based on P-tau217. However, false positives remain a concern, particularly when applying externally derived cutoffs that exhibited lower specificity which could have led to inappropriate treatment of Aβ-negative participants. Future research should focus on prospective validation of P-tau217 cutoffs to enhance their generalizability and inform standardized treatment decision-making across diverse populations.

**Supplementary Information:**

The online version contains supplementary material available at 10.1186/s13195-024-01521-9.

## Background

Access to novel disease modifying treatments (DMTs) for Alzheimer's disease (AD) hinges upon efficient detection of cerebral amyloid-β (Aβ) pathology in patients with early-stage disease [1, 2]. The recent Food and Drug Administration (FDA) approval of amyloid-lowering immunotherapies, such as aducanumab and lecanemab, necessitates the optimization of screening techniques for Aβ pathology, as biomarker confirmation of Aβ status is required prior to treatment [3, 4]. This important requirement represents a major barrier to treatment access for many patients due to current reliance on invasive and expensive positron emission tomography (PET) or cerebrospinal fluid (CSF) testing [5], and may compound existing racial and ethnic inequalities in AD treatment [6–9]. The development of a minimally invasive and cost-effective screening method to facilitate early detection and intervention is, therefore, of high clinical importance in expanding equitable access to new and forthcoming treatments for early AD [1, 10].

While Aβ-PET/CSF testing remains the standard-of-care, blood biomarkers have been extensively investigated and have been shown to accurately detect cerebral Aβ pathology in large observational cohort studies [10]. Among them, plasma tau phosphorylated at threonine-217 (P-tau217) has emerged as the most promising, with data from the Swedish BioFINDER study and others demonstrating high accuracy for prediction of Aβ status [11–14]. While the majority of this work has focused on using a single P-tau217 cutoff to define Aβ status (referred to here as the ‘one-cutoff’ approach), a number of assay and patient-dependent factors may contribute to test–retest variability and can lead to false positives and negatives [15]. Emerging data suggests that using a tiered ‘two-cutoff’ approach can partly circumvent this issue by defining a borderline or ‘gray zone’ of P-tau217 values that merit confirmatory testing [16, 17], and theoretically expedited eligibility screening when applied to potential DMT candidates from BioFINDER-2 in a recent publication by Mattsson-Carlgren et al*.* [17]. While these findings are promising, data are limited in clinical populations who are actively seeking treatment with DMTs, and there is a need to explore the generalizability of cutoffs between cohorts to represent how these tests are likely to be used in clinical practice.

Therefore, we sought to address these knowledge gaps by examining the diagnostic performance of P-tau217 at the Butler Hospital Memory & Aging Program (MAP). We hypothesized that plasma P-tau217 would predict Aβ positivity with high diagnostic accuracy when assessing eligibility for treatment with aducanumab. To confirm the usefulness of one- and two-cutoff approaches to P-tau217 interpretation in a relevant clinical context, we established cutoffs in a cohort of Butler MAP Aβ-positive and -negative controls of mixed cognitive status (n = 50), and then cross-validated these cutoffs in a separate cohort that included patients who subsequently received aducanumab alongside Aβ-positive and -negative controls with mild cognitive impairment (MCI) or mild dementia with no contraindications to aducanumab according to the Appropriate Use Recommendations (n = 50) [3]. We additionally compared diagnostic performance of these cutoffs to a matched sample of BioFINDER-2 participants with MCI or mild dementia (n = 50), as well as the previously published cut-offs identified by Mattsson-Carlgren et al*.* [17] in predicting Aβ status in all BioFINDER-2 participants deemed potentially eligible for DMTs.

Our findings highlight the promise of P-tau217 in expediting DMT eligibility determination, while also addressing key challenges that we address with site-specific cutoffs and comparison of interpretation strategies to handle intermediate P-tau217 values. Our findings, established in memory clinic patients seeking treatment with aducanumab, the first FDA-approved DMT, mark an important translational step towards expanding access to current and future DMTs, a key priority for the field that is expected to improve clinical outcomes for patients living with early AD.

## Methods

### Study design

We conducted a retrospective, cross-sectional, diagnostic cohort study of Butler Alzheimer’s Prevention Registry participants [18]. We included participants who began treatment with aducanumab in our memory clinic between June 16, 2021 and August 1, 2022, alongside additional Aβ-positive and -negative controls who were enrolled in the Registry Biobanking Substudy. We included all participants with MCI or mild-moderate dementia with known Aβ status in our analysis, including all participants who received aducanumab treatment and those who did not. To achieve sufficient sample size for model training, we also included Aβ-positive and negative controls with a lookback period of 3 years. Participants were then divided into Butler MAP Training and Test Cohorts according to the following criteria. The Training Cohort (n = 50) included all preclinical Aβ-positive and negative controls (n = 37) alongside untreated MCI or mild-moderate dementia cases (n = 13). The Test Cohort (n = 50) included all aducanumab-treated cases (n = 25) alongside randomly selected Aβ-positive and -negative controls with MCI or mild dementia (n = 25). To reflect the high prevalence of Aβ-positivity in our tertiary memory clinic setting, controls were selected to achieve an overall prevalence of ~ 70% Aβ-positivity in the total sample.

### Butler MAP participants

Registry participants were selected for aducanumab treatment by their memory clinic physician based on clinical judgement according to the Appropriate Use Recommendations, as previously described [3, 19]. To determine eligibility, the standard work-up included a full history, informant report, cognitive screening (Mini-Mental State Examination [MMSE], Montreal Cognitive Assessment [MoCA]), complete physical exam, general laboratory work-up, brain magnetic resonance imaging (MRI), and Aβ-PET/CSF testing. Neuropsychological testing was performed by a clinical neuropsychologist on a case-by-case basis for clinical purposes. A board-certified neurologist or geriatric psychiatrist was responsible for determining diagnosis, and for those found eligible, treatment decisions were made collaboratively with patients and families after careful informed consent. General data collection protocols for the Butler Alzheimer’s Prevention Registry are listed in the Supplementary Methods and have been previously published [18–20]. Study participation was not required to receive treatment with aducanumab. All participants provided written informed consent to sharing of deidentified data and biospecimens. Local study procedures were approved by the Butler Hospital Institutional Review Board (IRB #2108–001, #1604–001) and were consistent with the Helsinki Declaration of 1975.

### BioFINDER-2 participants

The BioFINDER-2 database was searched to generate a separate BioFINDER-2 Training Cohort (n = 50) containing participants with MCI or mild dementia. BioFINDER-2 participants were selected to approximately match to the Butler MAP Test Cohort on group-level clinical characteristics (Age, APOE-ε4 status, MMSE, Clinical Diagnosis, and Aβ positivity) irrespective of P-tau217 levels. Selection was blinded to participant-level data from the Butler MAP Test Cohort. Procedures for the Swedish BioFINDER-2 Study (NCT03174938) procedures were approved by the Regional Ethics Committee in Lund, Sweden. Details of these procedures have been described elsewhere and are summarized in the Supplementary Methods [11].

### Apolipoprotein E (APOE) genotyping

Participants underwent an optional cheek swab to collect epithelial cells for genotyping. Sequences were amplified using primers corresponding to each allele with the Genomadix PCR method as previously described [20].

### Plasma P-tau217 quantification

Laboratory procedures were identical for BioFINDER-2 and Butler MAP participants. Briefly, trained technicians drew whole venous blood (20 mL) into 10 ml EDTA draw tubes. Blood samples were centrifuged at 2,000 g for 15 min, then the plasma fraction was aliquoted into 1 mL polypropylene cryovials and stored at -80 °C. P-tau217 analysis was conducted in a single batch for Butler MAP and a separate batch for BioFINDER-2, respectively, by the Hansson laboratory at Lund University, Sweden, using the Mesoscale Discovery (MSD) platform in a blinded fashion as previously described [11]. The assay was calibrated using a recombinant tau (4R2N) protein that was phosphorylated in vitro and characterized by mass spectrometry [11, 21]. Plasma samples, after thawing on ice, were lightly vortexed and centrifuged at 2,000 g for 10 min. Plasma was then diluted 1:2 with sample buffer containing a heterophilic blocking reagent. MSD small-spot streptavidin-coated plates were prepared by blocking with 3% BSA in DPBS. After washing, biotinylated-IBA493, a capture antibody, was added to the wells for incubation. Following another wash, 50 μl of diluted plasma sample or calibrator was added to each well for a two-hour incubation. After this, the plates were washed, and the SULFO-tagged E2 (anti-Tau) detection antibody was introduced. Read buffer was applied and P-tau217 levels subsequently quantified using the MSD SQ120.

### Reference standards

In Butler MAP participants, we determined Aβ status using clinically available tests representing the standard-of-care for patients in the United States. Aβ-PET imaging was performed using FDA-approved radiotracers (^18^F-florbetapir or ^18^F-florbetaben), with positive or negative results determined by expert clinical visual read as previously described [22, 23]. For CSF analysis, lumbar puncture (LP) was performed and CSF concentrations of P-tau181, total tau and Aβ_42_ were measured using validated, clinically available immunoassays performed by Mayo Clinic Laboratories (Rochester, MN) or Athena Diagnostics (Marlborough, MA). CSF analysis was performed to calculate the P-tau181/Aβ_42_ ratio by Mayo Clinic Laboratories (Aβ positivity defined using a laboratory-specific cutoff of P-tau181/Aβ_42_ ≥ 0.028) or P-tau181 with Aβ_42_/total-tau index (ATI, Athena Diagnostics) using the ADMark® assay (Aβ positivity defined using laboratory-specific cutoffs as ATI < 1 and P-tau-181 > 61 pg/mL) [24]. CSF analysis was performed on a case-by-case basis as part of routine clinical care, with Aβ status reported by the laboratory based on these cutoffs which are in line with previously published data and recommended clinical guidelines [24–26]. BioFINDER-2 participants were defined as Aβ positive using the CSF Aβ_42_/_40_ ratio (cutoff < 0.08) as previously described (see Supplementary Methods) [11].

### Statistical analysis

All analyses were conducted in R (Version 4.2.2) using the pROC and caret packages by a blinded investigator. Receiver operator characteristic (ROC) analysis was employed to calculate the area under the curve (AUC) of the model, along with sensitivity, specificity, and positive/negative predictive values (PPV, NPV) at pre-specified cutoffs (Youden, 90% Sensitivity, 90% Specificity) that are consistent with the literature, using Aβ-PET/CSF testing as the standard of truth [17]. Participants in each cohort were categorized using one- and two-cutoff approaches. The one-cutoff approach assigned participants Aβ positive/negative status based on the Youden cutoff from the training data. Using the two-cutoff approach, participants were classified as negative, intermediate, or positive based on 90% Sensitivity or Specificity cutoffs. Those with intermediate P-tau217 values were handled using 'inclusive' and 'exclusive' strategies; the former assigned statuses based on known Aβ-PET/CSF results, while the latter excluded such participants from analysis. Confusion matrices for each strategy were generated using the caret package. Accuracy estimates and AUCs are reported with 95% confidence intervals (CI); accuracy was compared to the no-information rate using the exact binomial test, with statistical significance defined as *p* < 0.05.

Additionally, logistic regression was performed to examine the prediction of Aβ status with plasma P-tau217, with adjustment for age, sex, APOE-ε4 allele frequency, MoCA score, clinical diagnosis, and timing of reference standard testing (expressed as months from blood draw and Aβ-PET/CSF testing). All participants had available P-tau217 or Aβ-PET/CSF testing data, obviating the need for imputation or removal of cases for the primary outcomes reported in our analysis. For the adjusted model, missing-at-random covariates were handled by multiple imputation by chained equations, detailed in Supplementary Table 1.

## Results

### Participant characteristics

Participants (n = 100) were subdivided into the Butler MAP Training (n = 50) and Test Cohorts (n = 50) (Table 1; Supplementary Table 1). Most participants in the Butler MAP Training Cohort were Aβ-positive (n = 32 [64%]) using Aβ-PET/CSF results as the reference standard (Table 1). To study how P-tau217 would be used clinically to confirm Aβ status prior to receiving a DMT, the Butler MAP Test Cohort (n = 50) was limited to participants with a clinical diagnosis of MCI (n = 29 [58%]) or mild dementia (n = 21 [42%]) and no contraindication to aducanumab according to the Appropriate Use Recommendations [3]. The majority of Test Cohort participants were Aβ positive (n = 38 [76%]) and half received subsequent treatment with aducanumab (n = 25 [50%]). Apart from the between-cohort difference in cognition (MoCA: median = 25 [IQR: 22–27] vs. 22 [IQR: 18–23], *p* < 0.001), expected given the inclusion of cognitively normal (CN) controls in the Training Cohort (n = 37 [74%]), there were no differences in participant demographics, Aβ positivity or APOE genotype (Table 1; Supplementary Table 1).

**Table 1:** Demographics and clinical characteristics

**Characteristic**	**Overall**, N = 100^*1*^	**Butler MAP Training Cohort,** N = 50^*1*^	**Butler MAP Test Cohort**, N = 50^*1*^	***P*-value**^**2**^
Age	71 (64, 75)	69 (65, 75)	71 (64, 75)	0.96
Sex, male	46 (46%)	20 (40%)	26 (52%)	0.32
Race				0.28
*White*	77 (97%)	44 (100%)	33 (94%)	
*Black*	1 (1.3%)	0 (0%)	1 (2.9%)	
*Mixed race*	1 (1.3%)	0 (0%)	1 (2.9%)	
*Not reported*	21	6	15	
Ethnicity				> 0.99
*Not Hispanic or Latino*	74 (97%)	42 (98%)	32 (97%)	
*Hispanic or Latino*	2 (2.6%)	1 (2.3%)	1 (3.0%)	
*Not reported*	24	7	17	
APOE-ε4^3^				0.86
*Non-carrier*	25 (28%)	11 (27%)	14 (30%)	
*Heterozygote*	51 (58%)	25 (61%)	26 (55%)	
*Homozygote*	12 (14%)	5 (12%)	7 (15%)	
*Unknown*	*12*	*9*	*3*	
Clinical diagnosis				< 0.001
*Cognitively normal*	*37 (37%)*	*37 (74%)*	*0 (0%)*	
*Mild cognitive impairment*	*36 (36%)*	*7 (14%)*	*29 (58%)*	
*Dementia*	*27 (27%)*	*6 (12%)*	*21 (42%)*	
MMSE, score	27 (25, 29)	28 (26, 29)	27 (24, 28)	0.013
MoCA, score	23 (19, 25)	25 (22, 27)	22 (18, 23)	< 0.001
Aβ positivity	70 (70%)	32 (64%)	38 (76%)	0.28

^1^ Median (IQR); n (%)

^2^ Wilcoxon rank sum test; Pearson's Chi-squared test

To additionally control for potential effects of between-cohort differences in cognition, and inform generalizability of cutoffs between studies, we also selected BioFINDER-2 participants with MCI or mild dementia (n = 50) to generate a second Training Cohort that was matched on age, APOE genotype, clinical diagnosis, MMSE and Aβ-positivity to the Butler MAP Test Cohort (Table 2).

**Table 2:** Clinical characteristics of matched BioFINDER-2 participants

**Characteristic**	**BioFINDER-2 Training Cohort**^**a**^**,** N = 50^b^
Age, years	72 (66, 75)
Sex, male	27 (54%)
APOE-ε4	
*Non-carrier*	*14 (28%)*
*Heterozygote*	*29 (58%)*
*Homozygote*	*7 (14%)*
*Not reported*	*0*
MMSE, score	26 (25, 27)
Clinical Diagnosis	
*Mild cognitive impairment*	*29 (58%)*
*Mild dementia*	*21 (42%)*
Aβ positivity^c^	38 (76%)

^a^Cohort of BioFINDER-2 participants that were randomly selected to match group-level demographics and clinical characteristics (Age, Sex, APOE-ε4 status, MMSE, Clinical Diagnosis, and Aβ positivity) in the Butler MAP Test Cohort, blinded to P-tau217 concentration

^b^n (%); Median (IQR)

### Diagnostic performance of plasma P-tau217

**Fig. 1:**
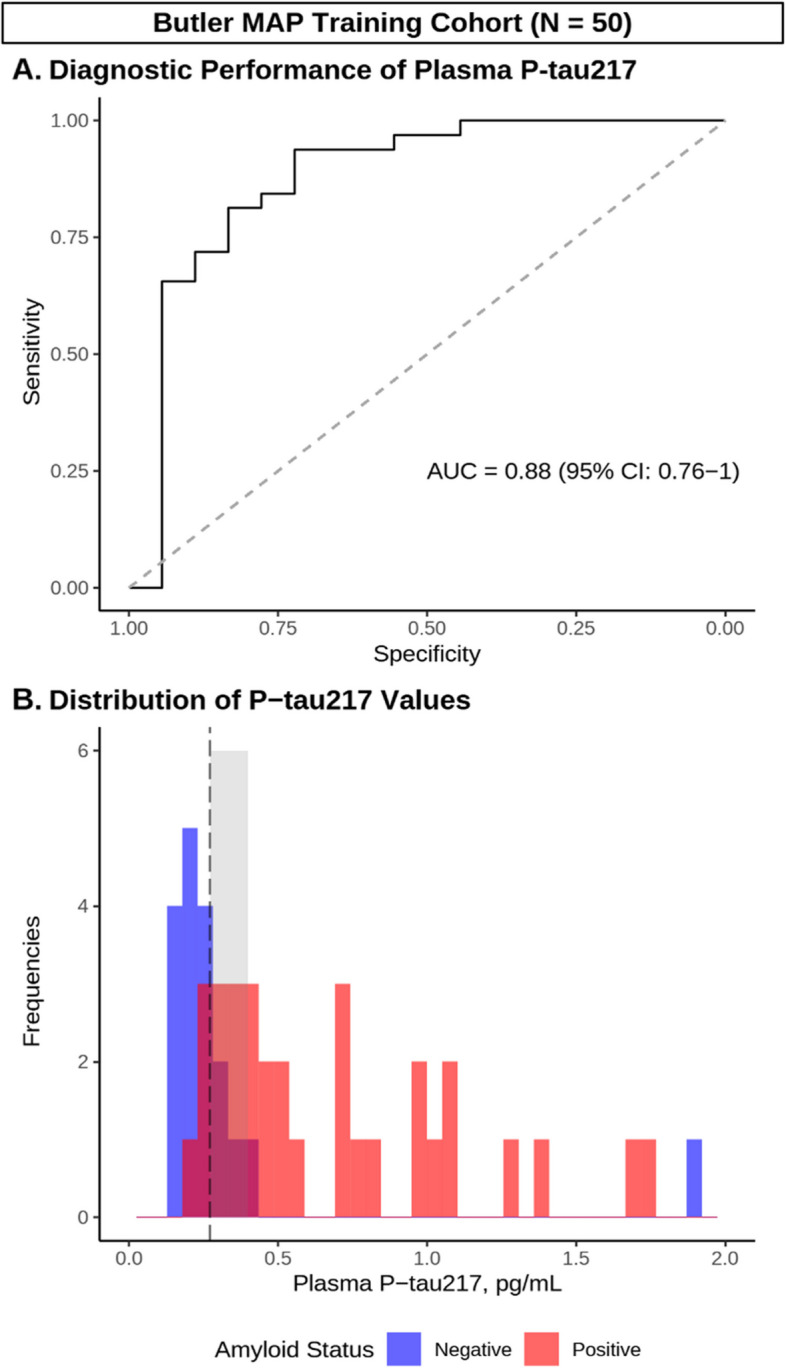
Development of P-tau217 cutoffs using site-specific training data. **A** ROC curve demonstrating diagnostic performance of P-tau217 in training data from Butler MAP cases/controls. **B** Histogram depicting the distribution of P-tau217 values (pg/mL) in Aβ negative (blue) and positive (red) participants, as well as Youden’s optimal cutoff (black dashed line) and the intermediate region (shaded gray). *N* = *50*

**Fig. 2:**
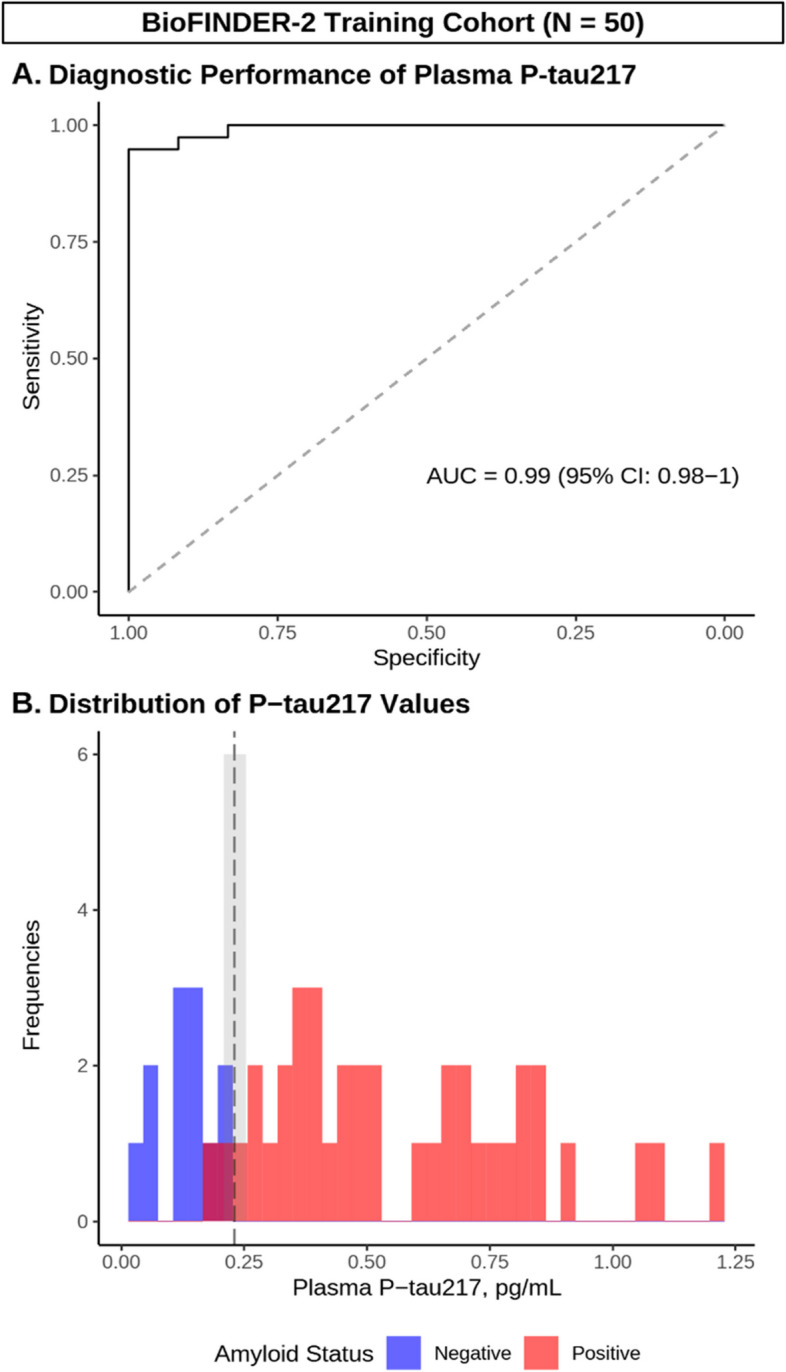
Development of P-tau217 cutoffs using external training data. **A** ROC curve demonstrating diagnostic performance of P-tau217 in cases/controls from BioFINDER-2. **B** Histogram depicting the distribution of P-tau217 values (pg/mL) in Aβ negative (blue) and positive (red) participants, as well as Youden’s optimal cutoff (black dashed line) and the intermediate region (shaded gray). *N* = *50*

To examine the diagnostic performance of plasma P-tau217, we first performed ROC analysis with confirmed Aβ status (by PET or CSF) as the outcome variable in the Butler MAP Training Cohort (Fig. 1). This analysis indicated that P-tau217 predicted Aβ status with an AUC of 0.88 (95% CI: 0.76 – 1). Similarly, ROC analysis in the BioFINDER-2 Training Cohort identified robust prediction of Aβ status with P-tau217 (AUC = 0.99 [95% CI: 0.98–1]) (Fig. 2).

In Butler MAP participants, adjusting for age, sex, APOE-ε4 genotype, MoCA score, clinical diagnosis, as well as timing and type of reference standard used (PET or CSF) did not attenuate the relationship between P-tau217 and Aβ positivity, with statistically significant independent effects observed for P-tau217 (*p* = 0.001), age (*p* = 0.002) and APOE-ε4 genotype (*p* = 0.019) (Supplementary Table 2). The adjusted model exhibited similar performance to P-tau217 alone in the Butler MAP Training Cohort (AUC = 0.91 [95% CI: 0.88–0.94]) (Supplementary Fig. 1). Taken together, these findings from two separate cohorts, as well as our adjusted model, consistently identified plasma P-tau217 as an independent predictor of Aβ positivity.

### Development of the one- and two-cutoff approaches

To first develop our approaches for individual-level biomarker interpretation, we used the Butler MAP Training Cohort to create two distinct models with prespecified plasma P-tau217 cutoff characteristics (Fig. 1, Panel B). For development of the one-cutoff model, we performed ROC analysis to identify the P-tau217 level which corresponds to the maximal Youden index. This approach identified an optimal one-cutoff of [P-tau217] ≥ 0.27 pg/mL, which in the Training Cohort predicted Aβ status with a diagnostic accuracy of 0.86 (95% CI: 0.73 – 0.94), *p* < 0.001; Fig. 1; Supplementary Table 3).

We then sought to improve prediction of intermediate P-tau217 values using the two-cutoff model, an alternative approach that stratifies participants using low and high cut-offs that are optimized for sensitivity and specificity, respectively. We first performed ROC analysis to identify a lower cutoff with a prespecified sensitivity ≥ 0.9, which corresponded to [P-tau217] < 0.273 pg/mL. Next, we identified a higher cutoff with a prespecified specificity ≥ 0.9, which corresponded to 0.399 pg/mL (prespecified sensitivity and specificity parameters were chosen based on the literature [17]). We then applied these cutoffs to stratify participants into three groups as follows: 1) Presumed Aβ-negative ([P-tau217] < 0.273 pg/mL), 2) Gray Zone ([P-tau217] = 0.273 – 0.399 pg/mL), and 3) Presumed Aβ-positive ([P-tau217] ≥ 0.399 pg/mL) (Fig. 1, Panel B).

Participants in groups 1 and 3 were classified based on their P-tau217 level, while participants falling in the Gray Zone (n = 12 [24%]) were instead either classified based on previously obtained Aβ-PET/CSF testing (‘inclusive’ approach, modeling a situation where these results triggered confirmatory testing for all participants) or removed from the analysis (‘exclusive’ approach, modeling a situation where these intermediate values were considered inconclusive, thus excluded from treatment). Applying the inclusive approach produced an overall diagnostic accuracy of 0.92 (95% CI: 0.81–0.98, *p* < 0.001), while using the exclusive approach produced a diagnostic accuracy of 0.89 (95% CI: 0.75–0.97, *p* < 0.001).

We repeated these analyses in the BioFINDER-2 Training Cohort, and found that the one-cutoff approach identified an optimal cutoff of [P-tau217] ≥ 0.231 pg/mL with a diagnostic accuracy of 0.94 (95% CI: 0.83 – 0.99, *p* < 0.001), while the two-cutoff approach (Presumed Negative: [P-tau217] < 0.209 or Presumed Positive: [P-tau217] ≥ 0.254 pg/mL) produced a diagnostic accuracy of up to 0.96 (95% CI: 0.86 – 1, *p* < 0.001; Fig. 2, Panel B). Detailed cutoff metrics for both Butler MAP and BioFINDER-2 Training Cohorts are presented in Supplementary Table 3.

In summary, our analysis found that while diagnostic performance of P-tau217 was similar between Butler MAP and BioFINDER-2 Training Cohorts, the cutoffs differed, with lower plasma [P-tau217] values favored in the BioFINDER-2 Training Cohort. Therefore, to assess the performance of these cutoffs and approaches when screening for DMT eligibility, we next sought to determine their performance when applied to the Butler MAP Test Cohort.

### Cross-validation of P-tau217 in the Butler MAP test cohort

**Fig. 3:**
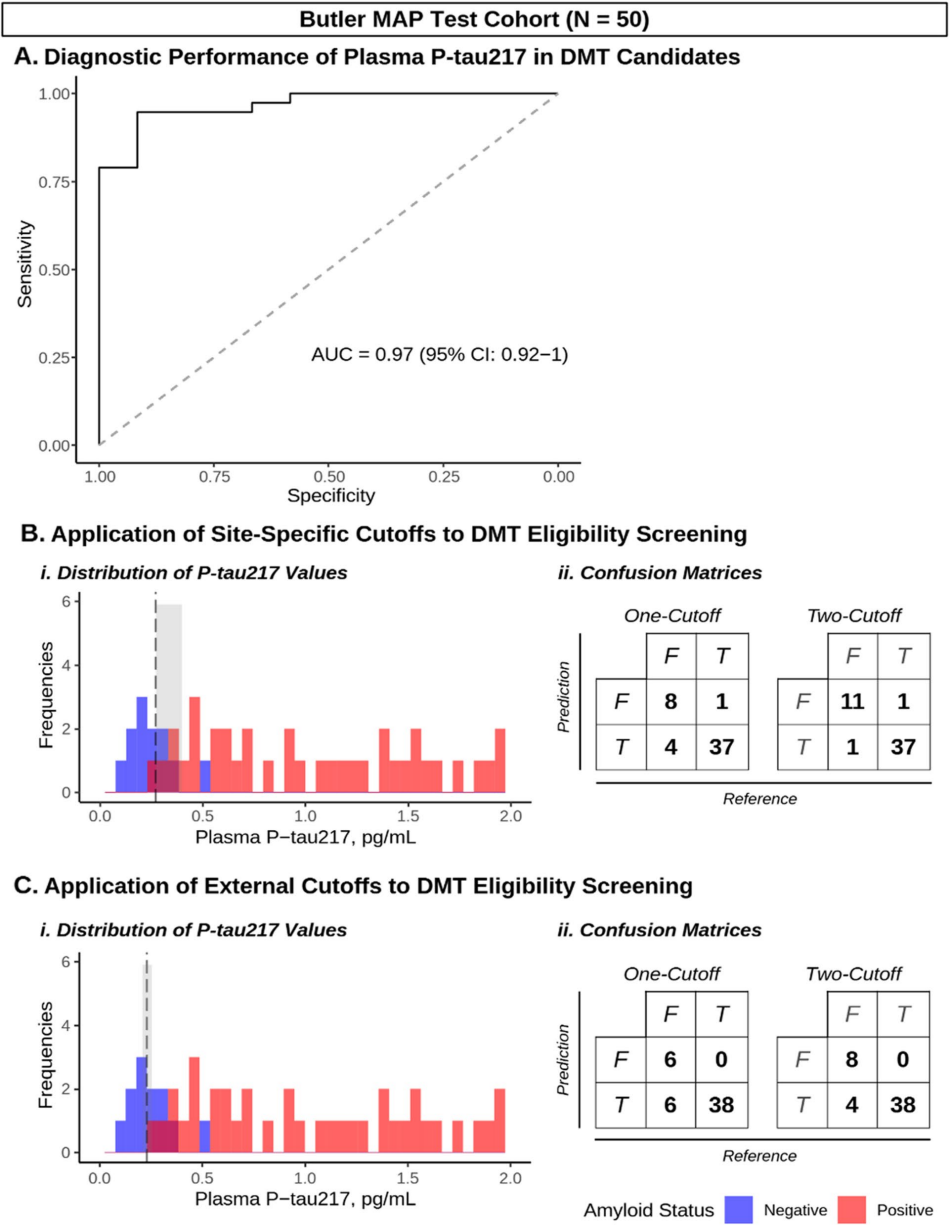
Diagnostic performance of one- and two-cutoffs for DMT eligibility screening. **A** ROC curve demonstrating diagnostic performance of P-tau217 in the Butler MAP Test Cohort. **B** Application of Butler MAP Training Cohort-derived (‘site-specific’) cutoffs to the Butler MAP Test Cohort, displayed as (i.) histogram and (ii.) confusion matrices for each approach, comparing P-tau217 (prediction) to Aβ-PET/CSF (reference). **C** Histogram and confusion matrices with ‘external’ cutoffs derived from matched BioFINDER-2 cases/controls. Histograms are color-coded by Aβ status: negative (blue) and positive (red), and overlayed with Youden’s optimal cutoff (black dashed line) and the intermediate region (shaded gray). *N* = *50*

To cross-validate our findings in potential DMT candidates from our memory clinic, we next examined the diagnostic performance of plasma P-tau217 in the Butler MAP Test Cohort (n = 50). ROC analysis demonstrated that P-tau217 predicted Aβ status in these participants, all potential DMT candidates (AUC = 0.97 [95% CI: 0.92 – 1]; Fig. 3, Panel A), which was also observed in the adjusted model (AUC = 0.99 [95% CI: 0.98 – 0.99]; Supplementary Fig. 2, Panel A). We then compared the one- and two-cutoff approaches using the ‘site-specific’ cutoffs generated in the Butler MAP Training Cohort, as well as those identified from the ‘external’ BioFINDER-2 Training Cohort to provide a comparison of cutoff generalizability.

Applying the site-specific cutoffs to the Butler MAP Test Cohort, we found that the one-cutoff approach demonstrated a diagnostic accuracy of 0.90 (95% CI: 0.78 – 0.96, *p* = 0.011; Table 3; Fig. 3, Panel B). The two-cutoff approach identified 6 (12%) of P-tau217 values falling within the Gray Zone, impacting 3 Aβ-positive and 3 Aβ-negative participants. When these gray zone cases were excluded from the analysis, the two-cutoff approach exhibited a diagnostic accuracy of 0.96 (0.86 – 1, *p* < 0.001); when these cases were included based on known Aβ-PET/CSF testing, the model had a diagnostic accuracy of 0.95 (95% CI: 0.84—0.99, *p* = 0.003; Table 3; Fig. 3, Panel B). Similar findings were observed with statistically significant diagnostic accuracy of one-cutoff and two-cutoff approaches in the adjusted model (all *p* < 0.001; Supplementary Table 4).

**Table 3:** Comparison of one and two-cutoffs for DMT eligibility screening

Model	Cutoffs	Total N	Specificity	Sensitivity	NPV	PPV	Accuracy (95% CI)^1^	*P*-value^2^
	**([P-tau217], pg/mL)**	**(Intermediate N)**						
**Site-Specific Cutoffs**								
One-cutoff	0.27	50	0.67	0.97	0.89	0.9	0.90 (0.78, 0.96)	0.011
Two-cutoff (Inclusive)	0.273, 0.399	50 (6)^3^	0.92	0.97	0.92	0.97	0.96 (0.86, 1)	< 0.001
Two-cutoff (Exclusive)	0.273, 0.399	44^4^	0.88	0.97	0.89	0.97	0.95 (0.84, 0.99)	0.003
**BioFINDER-2 Cutoffs**								
One-cutoff	0.231	50	0.5	1	1	0.86	0.88 (0.76, 0.95)	0.028
Two-cutoff (Inclusive)	0.209, 0.254	50 (5)^5^	0.67	1	1	0.9	0.92 (0.81, 0.98)	0.003
Two-cutoff (Exclusive)	0.209, 0.254	45^6^	0.5	1	1	0.9	0.9111 (0.79, 0.98)	0.079

^1^Accuracy is reported with 95% CI

^2^*P*-value: accuracy of model prediction of cerebral Aβ status compared to the no information rate

^3^BioFINDER-2 cutoffs identified 1 Aβ positive and 4 Aβ negative participants in the intermediate “gray zone” (5/50 = 10%)

^4^Remaining sample size after removal of 5 intermediate cases

^5^Site-specific cutoffs identified 3 Aβ positive and 3 Aβ negative participants in the intermediate “gray zone” (6/50 = 12%)

^6^Remaining sample size after removal of 6 intermediate cases

In comparison, applying the externally derived BioFINDER-2 cutoffs had a diagnostic accuracy of 0.88 (0.76 – 0.95, *p* = 0.028) using the one-cutoff approach, and an accuracy of 0.92 (95% CI: 0.81—0.98, *p* = 0.003) or 0.91 (0.79 – 0.98, *p* = 0.079) when using the two-cutoff approach with inclusive or exclusive handling of Gray Zone cases, respectively (n = 5 [10%]; 1 Aβ-positive and 4 Aβ-negative; Table 3; Fig. 3, Panel C). As an additional control, we also tested previously published cutoffs from an unmatched sample of BioFINDER-2 participants, which did not achieve statistically significant diagnostic accuracy, highlighting the dependency of these models on cutoff selection, regardless of the interpretation strategy used (Supplementary Table 5, Supplementary Fig. 3).

### Potential impact of P-tau217 screening on DMT eligibility

**Fig. 4:**
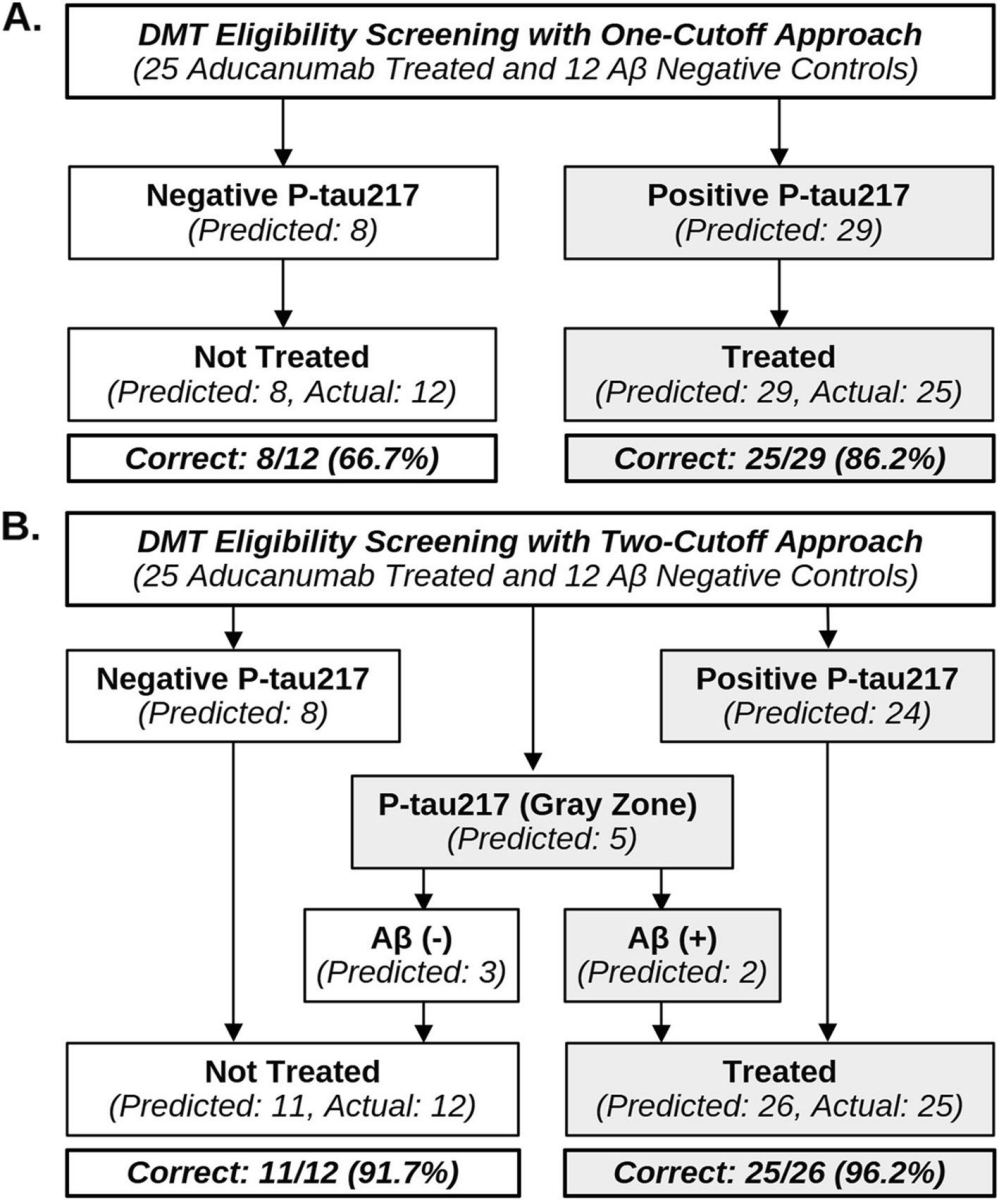
Flow-chart comparing DMT eligibility screening strategies. Analysis of predicted versus actual treatment decisions had P-tau217 been used to determine Aβ positivity in Butler MAP participants treated with aducanumab (n = 25), alongside Aβ negative controls (n = 12). **A** One-cutoff predictions compared to actual treatment decisions. **B** Two-cutoff predictions compared to actual treatment decisions. %Correct is calculated by comparing model predictions to the clinical decision to treat with aducanumab based on Aβ-PET/CSF testing. *N* = *37*

To explore how treatment decisions would have changed had P-tau217 been used to determine Aβ status in lieu of Aβ-PET/CSF testing, we then modeled how classifying participants based on P-tau217 screening would have affected treatment eligibility compared to actual treatment decisions. To further specify our findings, we limited this exploratory analysis to Aβ-positive participants who were subsequently treated with aducanumab (n = 25) and Aβ-negative controls (n = 12) who did not receive treatment, but could have been impacted by false positive results had P-tau217 been used to determine Aβ status (Fig. 4).

Applying the site-specific one-cutoff approach, we found that up to 25/25 (100%) of aducanumab-treated participants would have been found DMT eligible based on P-tau217 positivity, but as many as 4/12 (33.3%) Aβ-negative controls would have also qualified for treatment due to elevated P-tau217. Using the two-cutoff approach, a total of 5/37 (13.5%) participants would have fallen into the Gray Zone (2 aducanumab-treated Aβ-positives and 3 Aβ-negative controls). Assuming concordance with known Aβ-PET/CSF results (3 CSF assays and 2 PET scans), had reflex testing been performed it may have resulted in up to 25/25 (100%) of aducanumab-treated participants being found eligible for treatment. Had these cases been excluded from treatment decisions, treatment would have been withheld from 2/25 (8%) of Aβ-positives. In either case, 1/12 (8.3%) of the Aβ-negatives would have been found to be eligible for treatment due to P-tau217 falling above the Gray Zone region.

Examining generalizability of cutoffs between sites and studies, we also provide analysis of aducanumab treatment decisions using cutoffs derived from matched and unmatched BioFINDER-2 participants (Supplementary Fig. 4) [17]. All supplementary tables and figures are available in the Additional Files.

## Discussion

The current study offers evidence supporting the utility of plasma P-tau217 as an accessible biomarker for cerebral Aβ pathology, which is essential to confirm prior to initiation of amyloid-lowering agents. While aducanumab has since been discontinued by the manufacturer, the potential clinical application of our findings remains highly relevant given the recent FDA approval of lecanemab [3, 4], as well as the positive phase 3 data for donanemab [27], where accurate biomarker-based screening is paramount for candidate selection. Our findings agree with previous studies in the Swedish BioFINDER cohort which have shown similarly high diagnostic accuracy of P-tau217 in predicting Aβ positivity in MCI and mild dementia populations [11–13, 15–17]. The cross-validation of specific P-tau217 cutoffs in our Training and Test Cohort demonstrates its internal consistency and reliability across clinical scenarios at our site, however cutoffs generated from matched BioFINDER-2 participants exhibited mixed performance, and those from previously published data failed to replicate in our cohort [17]. Provided that these challenges in generalizability of cutoffs can be surmounted [10], P-tau217 could significantly enhance the scalability of DMT programs such as ours, paving the way for broader treatment accessibility in primary care and other non-tertiary settings.

These findings are timely given the anticipated *Revised Criteria for Diagnosis and Staging of AD* from the Alzheimer’s Association Work Group, which at present proposes that elevated plasma P-tau217 is sufficient to fulfill Core 1 criteria for A (amyloidosis) and T1 (secreted phosphorylated tau fragments) for a biomarker diagnosis of AD [28]. However, Core 2 criteria including tau tangle burden (T2) is not fully captured by P-tau217 and may be an important factor in predicting response to amyloid-lowering treatments, as seen in the recently published phase 3 data for donanemab [27]. A recent study identified CSF microtubule-binding region of tau containing the residue 243 (MTBR-tau43) as a novel fluid biomarker of tau tangle pathology, holding potential alongside P-tau217 and other biomarkers for detailed molecular staging of AD, although more work is needed to examine its performance as a more accessible blood test [29]. If successful, this methodological innovation would represent a major step forward in the biomarker field, potentially shaping future personalized medicine approaches to DMTs.

Our retrospective analysis indicates that P-tau217 is a promising tool to determine DMT eligibility for individuals seeking treatment with aducanumab, and our comparison of cutoffs and strategies in a cohort of patients subsequently treated with aducanumab marks an important translational step towards providing clinical guidance on the use of specific blood biomarker interpretation strategies at the level of an individual patient [30]. Our data suggest that the use of two-cutoffs to stratify patients is feasible in the memory clinic setting, and may reduce false positives to more effectively discriminate AD from non-AD cases. Rather than replacing PET/CSF tests entirely, this more nuanced approach addresses the diagnostic uncertainty in borderline cases by providing standardized guidance to target confirmatory testing to this group. While we relied on a single reference method for our analysis, removing the intermediate-range cases from treatment decisions also proved to be an effective approach. Alternatively, the use of targeted CSF testing to handle intermediate-range P-tau217 is supported by two recently published studies which showed that showed enhanced prediction of amyloid PET status using P-tau217 with reflex CSF confirmatory testing [16, 17]. Based on these results, it appears that the two-cutoff strategy could be a viable alternative for reducing reliance on PET/CSF testing when determining DMT eligibility. However, more work is needed to assess how clinical outcomes may change when these approaches are used to prospectively select patients for treatment.

Importantly, our study identified higher optimal cutoffs than the recent paper by Mattsson-Carlgren et al*.* [17], despite harmonized blood collection protocols, centralized measurement of P-tau217 levels, similar analytic approaches and large effect sizes for the prediction of amyloid status [17]. This could be due to differences in the inclusion of preclinical AD in the Butler MAP Training Cohort or test–retest variability in immunoassay performance, although the adjusted model and the cutoffs generated in the matched BioFINDER-2 Training Cohort argue against these explanations. Ongoing efforts by our laboratory and others to develop and refine standardized calibrators may help to further reduce inter-assay variability observed with immunoassay techniques [1]. Avenues for future research include examining prediction models that control for specific comorbidities and medications that may influence P-tau217 levels [15, 31, 32], as well as exploration of as-yet unknown lifestyle factors (i.e. diet, exercise, sleep, stress) and differing prevalence of AD that could impact choice of cutoff for a given population. Given the strong internal validity between training/test cohorts our study and the analysis by Mattsson-Carlgren et al*.* [17], and the differences in optimal cutoffs that were observed between these studies, we continue to recommend using site-specific cutoffs when considering implementation of P-tau217 for clinical use, as specific cutoffs are not yet generalizable.

While our study's findings are promising, they must be interpreted within the context of some important limitations. The retrospective, cross-sectional design limits our ability to compare P-tau217 with multiple measures of Aβ or tau status, and additionally limits our ability to assess treatment outcomes when P-tau217 is used to detect Aβ positivity compared to standard approaches. Although we have previously published data on a subset of participants who experienced amyloid-related imaging abnormalities (ARIA), treatment decisions were made based on Aβ-PET/CSF results in line with the Appropriate Use Recommendations for aducanumab [3, 19]. While our sample included all registry participants treated with aducanumab in our clinic (and are generally reflective of those who traditionally participate in DMT trials at our site), the potential for ascertainment bias and our limited sample size likely reduces generalizability compared to other, larger studies [17]. Furthermore, this cohort is not demographically diverse and does not fully represent the general population at risk for AD, a problem for research on blood biomarkers, DMTs and the field more broadly [6, 7]. There may be important fluid and imaging biomarker-related interactions with self-reported race, social determinants of health, medical comorbidities and APOE genotype that remain understudied and not captured by our analysis [33–36].

Future prospective research should seek to identify and address remaining barriers to routine clinical use with a focus on diverse populations in non-tertiary care settings. This is essential, especially in the context of efforts to expand treatment to traditionally underserved groups. Surmounting these remaining hurdles will require coordinated efforts between clinical sites to assess the precision and reliability of P-tau217 measurements when conducted on an individual basis, as opposed to batch testing, to ensure consistent and accurate results in clinical practice given the potential for inter-assay variability [1]. We must also move to assess dissemination and implementation-based strategies to improve communication of advances in the field to community healthcare providers and patients, with the goal of improving AD diagnosis and treatment in equitable and sustainable ways.

## Conclusions

This study substantiates the use of plasma P-tau217 as a viable biomarker for DMT eligibility screening, with potential significant impacts on clinical practice. The two-cutoff strategy presents an innovative method to refine biomarker analysis for the purpose of determining treatment eligibility or need for additional confirmatory testing. These findings underscore the challenge of applying specific cutoffs across studies and populations. Future research should aim to develop standardized cutoffs using samples drawn from multiple sites, and investigate broader clinical applications of P-tau217 and other blood biomarkers.

### Supplementary Information

Additional file 1: Supplementary Methods.

Additional file 2: Supplementary Table 1. Extended demographics and clinical characteristics.

Additional file 3: Supplementary Table 2. Logistic regression model for prediction of Aβ positivity.

Additional file 4: Supplementary Fig. 1. Development of adjusted cutoffs using Butler MAP training data.

Additional file 5: Supplementary Table 3. Diagnostic performance of individual P-tau217 cutoffs by cohort.

Additional file 6: Supplementary Fig. 2. Diagnostic performance of adjusted cutoffs for DMT eligibility screening.

Additional file 7: Supplementary Table 4. Diagnostic performance of adjusted cutoffs for DMT eligibility screening.

Additional file 8: Supplementary Table 5. Diagnostic performance of previously published cutoffs for DMT eligibility screening.

Additional file 9: Supplementary Fig. 3. Diagnostic performance of previously published cutoffs for DMT eligibility screening.

Additional file 10: Supplementary Fig. 4. DMT eligibility flow charts using BioFINDER-2 cutoffs.

## Data Availability

No datasets were generated or analysed during the current study.
