# Supplementary material for: Clinical application of plasma P-tau217 to assess eligibility for amyloid-lowering immunotherapy in memory clinic patients with early Alzheimer’s disease
Source: Alzheimers Res Ther. 2024 Jul 6;16:154. doi: 10.1186/s13195-024-01521-9 (PMC11227160; doi:10.1186/s13195-024-01521-9)
Supplement: Supplementary file 1 — Additional file 1: Supplementary Methods. [file 13195_2024_1521_MOESM1_ESM.docx]

**(Additional File 1)**

**Supplementary Methods**

**Butler Alzheimer’s Prevention Registry Procedures**

To identify potential study participants, our study team reviewed deidentified participant data (‘registrants’) from the Butler Alzheimer’s Prevention Registry, a database of over 3,000 community-dwelling adults in southern New England who are aged 40 to 85 and express potential interest in future research studies (Lee et al., 2017). Referral sources include the Butler Hospital Memory Disorders Clinic, community-based self or physician referrals, as well as clinical trials offered at our site. Target groups include healthy controls without any cognitive diagnosis alongside MCI or AD dementia cases. Registrants may be invited to participate in clinical trials as well as additional optional sub-studies, including APOE risk disclosure and banking of deidentified biospecimens (plasma, serum, CSF) (Lee et al., 2023). The registry database is updated regularly following any clinic or research visits at our site by review of electronic medical record data by research staff. The database is periodically reviewed to ensure data quality and accuracy of contact information, demographic data, cognitive assessments, clinical diagnosis, and any available laboratory/imaging results (e.g. Aβ status, APOE genotype).

**BioFINDER-2 procedures**

The study included 50 patients with MCI and AD dementia from the Swedish BioFINDER-2 study (NCT03174938) (Palmqvist et al., 2020). Participants were classified as having MCI if they performed worse than -1.5 SD in any cognitive domain according to test norms adjusted for age and education. The neuropsychological battery covered the domains attention/executive function (Trail Making Test A and B and Symbol Digit Modalities Test), verbal ability (verbal fluency animals and the 15-word short version of the Boston Naming Test), memory (10-word delayed recall from the Alzheimer’s Disease (AD) Assessment Scale [ADAS]), and visuospatial function (incomplete letters and cube analysis from the Visual Object and Space Perception battery [VOSP]). A clinical diagnosis of AD dementia was based on the DSM-5 criteria for major neurocognitive disorder due to AD and positive Aβ status in agreement with the most recent NIA-AA criteria for AD (Jack *et al.,* 2018). Further details of inclusion/exclusion criteria have been previously described (Palmqvist et al., 2020). The BioFINDER-2 study were approved by the Regional Ethics Committee in Lund, Sweden and all participants provided written informed consent.

CSF and blood samples were drawn in the morning while participants were not necessarily non-fasting. Blood was collected in six K2-EDTA-plasma tubes and centrifuged at 2000g, +4°C for 10 minutes. Following centrifugation plasma was aliquoted into 1.5-ml polypropylene tubes (1 ml per tube) and stored at −80°C. CSF was obtained by lumbar puncture and stored at −80°C in polypropylene tubes following the Alzheimer’s Association flow chart for lumbar puncture and CSF sample processing. CSF Aβ_40_ and Aβ_42_ were measured using Roche Elecsys immuno-assay and NeuroToolKit, respectively CSF Aβ status (negative/positive) was determined using the CSF Aβ_42/40_ ratio based on a previously described threshold of 0.08 (Quadalti et al., 2023).

**References**

Jack CR, Jr., Bennett DA, Blennow K, et al. NIA-AA Research Framework: Toward a biological definition of Alzheimer's disease.

Alzheimers Dement. 2018;14(4):535-562. doi:10.1016/j.jalz.2018.02.018

Lee A, Alber J, Monast D, Menard W, Tang T, Bodge C, et al. The Butler Alzheimer’s Prevention Registry: Recruitment and Interim

Outcome. In: Alzheimer’s & Dementia. 2017. p. 622–3.

Lee AKW, Collier MK, Thompson LI, Popescu D, Arthur E, Correia S, et al. The Effects of Subjective Cognitive Decline on APOE

Genotype Disclosure in the Butler Hospital Alzheimer’s Prevention Registry. J Prev Alz Dis. 2023 Feb 1;10(2):152–61.

Palmqvist S, Janelidze S, Quiroz YT, et al. Discriminative Accuracy of Plasma Phospho-tau217 for Alzheimer Disease vs Other

Neurodegenerative Disorders. JAMA. 2020;doi:10.1001/jama.2020.12134

Quadalti C, Palmqvist S, Hall S, et al. Clinical effects of Lewy body pathology in cognitively impaired individuals. Nat Med.

2023;29(8):1964-1970. doi:10.1038/s41591-023-02449-7
