# Supplementary material for: Clinical application of plasma P-tau217 to assess eligibility for amyloid-lowering immunotherapy in memory clinic patients with early Alzheimer’s disease
Source: Alzheimers Res Ther. 2024 Jul 6;16:154. doi: 10.1186/s13195-024-01521-9 (PMC11227160; doi:10.1186/s13195-024-01521-9)
Supplement: Supplementary file 2 — Additional file 2: Supplementary Table 1. Extended demographics and clinical characteristics. [file 13195_2024_1521_MOESM2_ESM.docx]

**(Additional File 2)**

| **Supplementary Table 1. Extended demographics and clinical characteristics.** | | | | | |
| --- | --- | --- | --- | --- | --- |
| **Characteristic** | | **Overall**,  N = 100*^1^* | **Training Cohort**,  N = 50*^1^* | **Test Cohort**,  N = 50*^1^* | **P-value^2^** |
| Age | | 71 (64, 75) | 69 (65, 75) | 71 (64, 75) | 0.96 |
| Sex, male | | 46 (46%) | 20 (40%) | 26 (52%) | 0.32 |
| Race | |  |  |  | 0.28 |
|  | *White* | *77 (97%)* | *44 (100%)* | *33 (94%)* |  |
|  | *Black* | *1 (1.3%)* | *0 (0%)* | *1 (2.9%)* |  |
|  | *Mixed race* | *1 (1.3%)* | *0 (0%)* | *1 (2.9%)* |  |
|  | *Not reported* | *21* | *6* | *15* |  |
| Ethnicity | |  |  |  | >0.99 |
|  | *Not Hispanic or Latino* | *74 (97%)* | *42 (98%)* | *32 (97%)* |  |
|  | *Hispanic or Latino* | *2 (2.6%)* | *1 (2.3%)* | *1 (3.0%)* |  |
|  | *Not reported* | *24* | *7* | *17* |  |
| APOE-ε4 | |  |  |  | 0.86 |
|  | *Non-carrier* | *25 (28%)* | *11 (27%)* | *14 (30%)* |  |
|  | *Heterozygote* | *51 (58%)* | *25 (61%)* | *26 (55%)* |  |
|  | *Homozygote* | *12 (14%)* | *5 (12%)* | *7 (15%)* |  |
|  | *Missing* | *12* | *9* | *3* |  |
| MMSE, score | | 27 (25, 29) | 28 (26, 29) | 27 (24, 28) | 0.013 |
|  | *Missing* | *9* | *9* | *0* |  |
| MoCA, score | | 23 (19, 25) | 25.0 (22, 27) | 22 (18, 23) | <0.001 |
|  | *Missing* | *13* | *9* | *4* |  |
| Clinical diagnosis | |  |  |  | <0.001 |
|  | *Cognitively normal* | *37 (37%)* | *37 (74%)* | *0 (0%)* |  |
|  | *Mild cognitive impairment* | *36 (36%)* | *7 (14%)* | *29 (58%)* |  |
|  | *Dementia* | *27 (27%)* | *6 (12%)* | *21 (42%)* |  |
| Type of Aβ testing | |  |  |  | <0.001 |
|  | *CSF only* | *29 (29%)* | *6 (12%)* | *23 (46%)* |  |
|  | *PET only* | *66 (66%)* | *41 (82%)* | *25 (50%)* |  |
|  | *Both* | *5 (5.0%)* | *3 (6.0%)* | *2 (4.0%)* |  |
| Timing of amyloid testing (months) | | -1 (-7, 0) | -2 (-13, 0) | 0 (-4, 0) | 0.023 |
| Aβ positivity | | 70 (70%) | 32 (64%) | 38 (76%) | 0.28 |
| *^1^* Median (IQR); n (%) | | | | | |
| *^2^* Wilcoxon rank sum test; Pearson's Chi-squared test | | | | | |
