# Supplementary material for: Clinical application of plasma P-tau217 to assess eligibility for amyloid-lowering immunotherapy in memory clinic patients with early Alzheimer’s disease
Source: Alzheimers Res Ther. 2024 Jul 6;16:154. doi: 10.1186/s13195-024-01521-9 (PMC11227160; doi:10.1186/s13195-024-01521-9)
Supplement: Supplementary file 3 — Additional file 3: Supplementary Table 2. Logistic regression model for prediction of Aβ positivity. [file 13195_2024_1521_MOESM3_ESM.docx]

**(Additional File 3)**

| **Supplementary Table 2. Logistic regression model for prediction of Aβ positivity.** | | | |
| --- | --- | --- | --- |
| **Characteristic** | **OR** | **95% CI** | **P-value** |
| P-tau217 (z) | 18.6 | 3.25, 107 | 0.001 |
| APOE-ε4 | 10.9 | 1.49, 79.3 | 0.019 |
| Age (z) | 6.3 | 2.03, 19.6 | 0.002 |
| Clinical diagnosis (MCI or dementia) | 6.13 | 0.59, 64.1 | 0.13 |
| MoCA (z) | 3.06 | 0.90, 10.4 | 0.073 |
| Type of amyloid testing (CSF) | 1.31 | 0.14, 12.3 | 0.8 |
| Timing of amyloid testing (z) | 0.87 | 0.25, 2.96 | 0.8 |
| Sex, male | 0.28 | 0.06, 1.32 | 0.11 |
| Data are reported for the combined training and test cohorts to maximize statistical power (n = 99). Missing data were handled by imputation, and continuous variables were converted to z-scores. Logistic regression was performed on variables of interest to predict cerebral Aβ status. Race/ethnicity was omitted due to insufficient sample size. To ensure model stability, we removed one statistical outlier of an Aβ-positive participant with markedly elevated P-tau217 level of 29.9 pg/mL (25 pg/mL on repeat testing). OR = Odds Ratio, CI = Confidence Interval. | | | |
