# Supplementary material for: Clinical application of plasma P-tau217 to assess eligibility for amyloid-lowering immunotherapy in memory clinic patients with early Alzheimer’s disease
Source: Alzheimers Res Ther. 2024 Jul 6;16:154. doi: 10.1186/s13195-024-01521-9 (PMC11227160; doi:10.1186/s13195-024-01521-9)
Supplement: Supplementary file 5 — Additional file 5: Supplementary Table 3. Diagnostic performance of individual P-tau217 cutoffs by cohort. [file 13195_2024_1521_MOESM5_ESM.docx]

**(Additional File 5)**

| **Supplementary Table 3. Diagnostic performance of individual P-tau217 cutoffs by cohort.** | | | | | | | |
| --- | --- | --- | --- | --- | --- | --- | --- |
| **Training Data** | **Cutoff Method** | **Cutoff [P-tau217]** | **Specificity** | **Sensitivity** | **Accuracy** | **NPV** | **PPV** |
|  |  |  |  |  |  |  |  |
| **Butler MAP Training Cohort, N = 50** | | | | | | | |
| Butler MAP Training Cohort | Youden cutoff (Balanced) | 0.27 | 0.72 | 0.94 | 0.86 | 0.87 | 0.86 |
| Butler MAP Training Cohort | Low cutoff (> 90% Sensitivity) | 0.273 | 0.72 | 0.91 | 0.84 | 0.81 | 0.85 |
| Butler MAP Training Cohort | High cutoff (> 90% Specificity) | 0.399 | 0.94 | 0.66 | 0.76 | 0.61 | 0.95 |
| **Butler MAP Test Cohort, N = 50** | | | | | | | |
| Butler MAP Training Cohort | Youden cutoff (Balanced) | 0.27 | 0.67 | 0.97 | 0.9 | 0.89 | 0.9 |
| Butler MAP Training Cohort | Low cutoff (> 90% Sensitivity) | 0.273 | 0.67 | 0.97 | 0.9 | 0.89 | 0.9 |
| Butler MAP Training Cohort | High cutoff (> 90% Specificity) | 0.399 | 0.92 | 0.87 | 0.88 | 0.69 | 0.97 |
| **BioFINDER-2 Training Cohort, N = 50** | | | | | | | |
| BioFINDER-2 Training Cohort | Youden cutoff (Balanced) | 0.231 | 1 | 0.95 | 0.96 | 0.86 | 1 |
| BioFINDER-2 Training Cohort | Low cutoff (> 90% Sensitivity) | 0.209 | 0.92 | 0.97 | 0.96 | 0.92 | 0.97 |
| BioFINDER-2 Training Cohort | High cutoff (> 90% Specificity) | 0.254 | 1 | 0.92 | 0.94 | 0.8 | 1 |
| **Butler MAP Test Cohort, N = 50** | | | | | | | |
| BioFINDER-2 Training Cohort | Youden cutoff (Balanced) | 0.231 | 0.5 | 1 | 0.88 | 1 | 0.86 |
| BioFINDER-2 Training Cohort | Low cutoff (> 90% Sensitivity) | 0.209 | 0.33 | 1 | 0.84 | 1 | 0.83 |
| BioFINDER-2 Training Cohort | High cutoff (> 90% Specificity) | 0.254 | 0.67 | 0.97 | 0.9 | 0.89 | 0.9 |
|  | | | | | | | |
