# Supplementary material for: Clinical application of plasma P-tau217 to assess eligibility for amyloid-lowering immunotherapy in memory clinic patients with early Alzheimer’s disease
Source: Alzheimers Res Ther. 2024 Jul 6;16:154. doi: 10.1186/s13195-024-01521-9 (PMC11227160; doi:10.1186/s13195-024-01521-9)
Supplement: Supplementary file 6 — Additional file 6: Supplementary Fig. 2. Diagnostic performance of adjusted cutoffs for DMT eligibility screening. [file 13195_2024_1521_MOESM6_ESM.docx]

**(Additional File 6)**


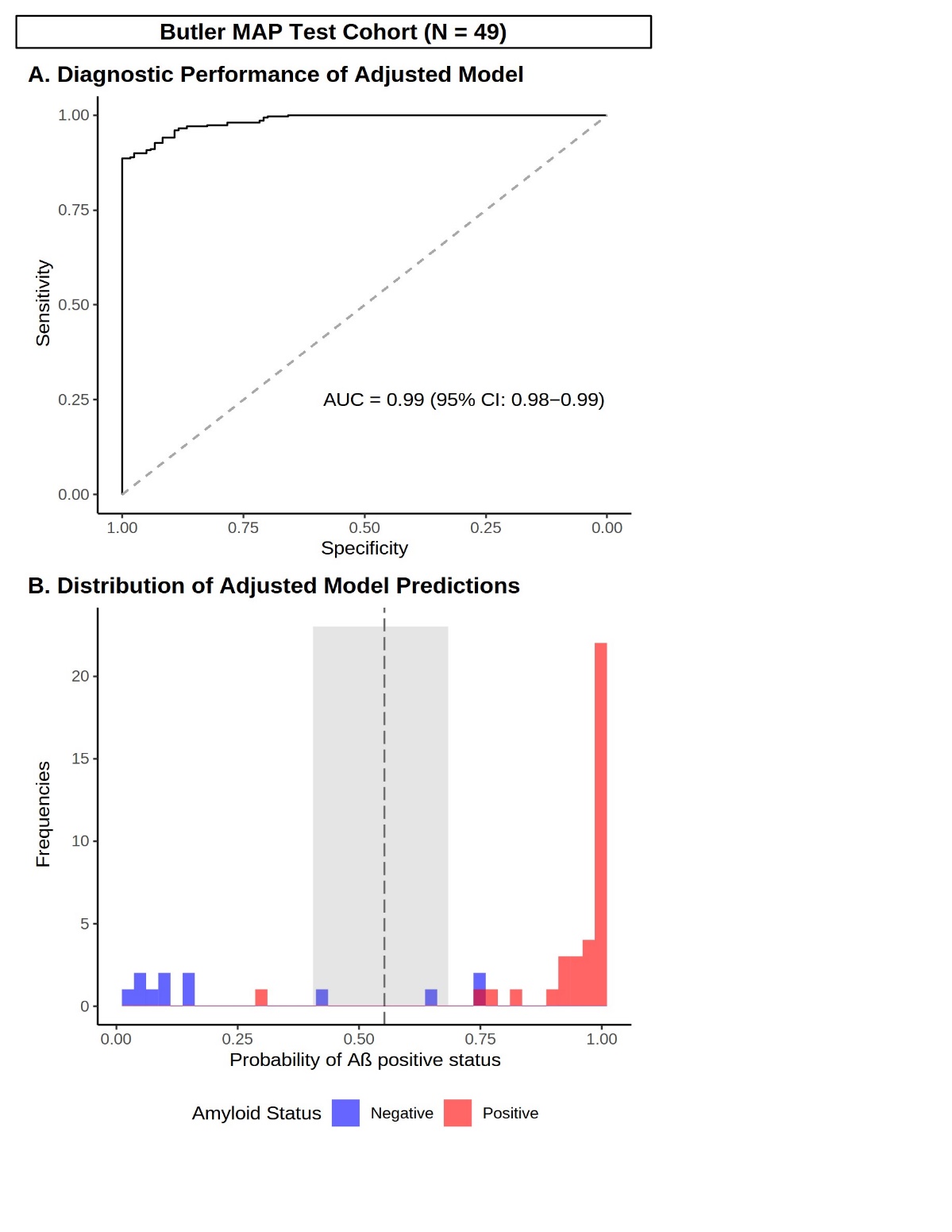


**Supplementary Figure 2. Diagnostic performance of adjusted cutoffs for DMT eligibility screening. (A)** ROC curve demonstrating diagnostic performance of a logistic regression model containing P-tau217 and all covariates of interest (age, APOE genotype, clinical diagnosis, cognition [MoCA], timing of plasma draw and type of reference standard [Aβ-PET/CSF]) in the test cohort. **(B)** Histogram depicting the distribution of the regression fitted values in Aβ positive (red) and negative (blue) participants, as well as Youden’s optimal cutoff (black dashed line) and the intermediate region (shaded gray). One outlier was removed, see *Supplementary Table 2* for individual predictors and explanation of outlier procedure*. N = 49.*
