# Supplementary material for: Clinical application of plasma P-tau217 to assess eligibility for amyloid-lowering immunotherapy in memory clinic patients with early Alzheimer’s disease
Source: Alzheimers Res Ther. 2024 Jul 6;16:154. doi: 10.1186/s13195-024-01521-9 (PMC11227160; doi:10.1186/s13195-024-01521-9)
Supplement: Supplementary file 7 — Additional file 7: Supplementary Table 4. Diagnostic performance of adjusted cutoffs for DMT eligibility screening. [file 13195_2024_1521_MOESM7_ESM.docx]

**(Additional File 7)**

| **Supplementary Table 4. Diagnostic performance of the adjusted model in the test cohort.** | | | | | | | | |
| --- | --- | --- | --- | --- | --- | --- | --- | --- |
| **Model^1^** | **Cutoffs (Probability)^2^** | **Total N**  **(Intermediate N)** | **Specificity** | **Sensitivity** | **NPV** | **PPV** | **Accuracy** | **p-value^4^** |
| One-cutoff | 0.552 | 49 | 0.78 | 0.97 | 0.90 | 0.93 | 0.93 (0.90, 0.95) | < 0.001 |
| Two-cutoff (Inclusive) | 0.406, 0.684 | 49 (2)^5^ | 0.85 | 0.99 | 0.97 | 0.95 | 0.96 (0.94, 0.97) | < 0.001 |
| Two-cutoff (Exclusive) | 0.406, 0.684 | 47^6^ | 0.83 | 0.99 | 0.97 | 0.95 | 0.95 (0.93, 0.97) | < 0.001 |
| ^1^Logistic regression model containing P-tau217 and all covariates of interest (age, APOE genotype, clinical diagnosis, cognition [MoCA], timing of plasma draw and type of reference standard [Aβ-PET/CSF]).  ^2^Cutoffs are reported as fitted probability values.  ^3^Accuracy is reported with 95% CI.  ^4^P-value: accuracy of model prediction of cerebral Aβ status compared to the no information rate.  ^5^Probability-based cutoffs identified 2 Aβ negative participants in the intermediate “gray zone” (2/49 = 4.08%).  ^6^Remaining sample size after removal of 2 intermediate cases. | | | | | | | | |
