# Supplementary material for: Clinical application of plasma P-tau217 to assess eligibility for amyloid-lowering immunotherapy in memory clinic patients with early Alzheimer’s disease
Source: Alzheimers Res Ther. 2024 Jul 6;16:154. doi: 10.1186/s13195-024-01521-9 (PMC11227160; doi:10.1186/s13195-024-01521-9)
Supplement: Supplementary file 8 — Additional file 8: Supplementary Table 5. Diagnostic performance of previously published cutoffs for DMT eligibility screening. [file 13195_2024_1521_MOESM8_ESM.docx]

**(Additional File 8)**

| **Supplementary Table 5. Diagnostic performance of previously published cutoffs for DMT eligibility screening.** | | | | | | | | |
| --- | --- | --- | --- | --- | --- | --- | --- | --- |
| **Model** | **Cutoffs^1^**  **([P-tau217], pg/mL)** | **Total N**  **(Intermediate N)** | **Specificity** | **Sensitivity** | **NPV** | **PPV** | **Accuracy^2^** | **p-value^3^** |
| One-cutoff | 0.22 | 50 | 0.33 | 1.00 | 1.00 | 0.83 | 0.84 (0.71, 0.93) | 0.121 |
| Two-cutoff (Inclusive) | 0.159, 0.219 | 50 (1)^4^ | 0.33 | 1.00 | 1.00 | 0.83 | 0.84 (0.71, 0.93) | 0.121 |
| Two-cutoff (Exclusive) | 0.159, 0.219 | 49 | 0.27 | 1.00 | 1.00 | 0.83 | 0.84 (0.71, 0.93) | 0.198 |
| ^1^Cutoffs were derived from Swedish BioFINDER-2 then applied to the Test Cohort (N = 50), see Mattsson-Carlgren et al ***.  ^2^Accuracy is reported with 95% CI.  ^3^P-value: accuracy of model prediction of cerebral Aβ status compared to the no information rate.  ^4^Analysis identified 2 Aβ negative participants in the intermediate “gray zone” (1/50 = 2.5%).  ^5^Remaining sample size after removal of 1 intermediate case. | | | | | | | | |

**Reference:**

Mattsson-Carlgren N, Collij LE, Stomrud E, Pichet Binette A, Ossenkoppele R, Smith R, et al. Plasma Biomarker Strategy for Selecting Patients With Alzheimer Disease for Antiamyloid Immunotherapies. JAMA Neurol. 2024 Jan 1;81(1):69–78.
