# Supplementary material for: Clinical application of plasma P-tau217 to assess eligibility for amyloid-lowering immunotherapy in memory clinic patients with early Alzheimer’s disease
Source: Alzheimers Res Ther. 2024 Jul 6;16:154. doi: 10.1186/s13195-024-01521-9 (PMC11227160; doi:10.1186/s13195-024-01521-9)
Supplement: Supplementary file 9 — Additional file 9: Supplementary Fig. 3. Diagnostic performance of previously published cutoffs for DMT eligibility screening. [file 13195_2024_1521_MOESM9_ESM.docx]

**(Additional File 9)**


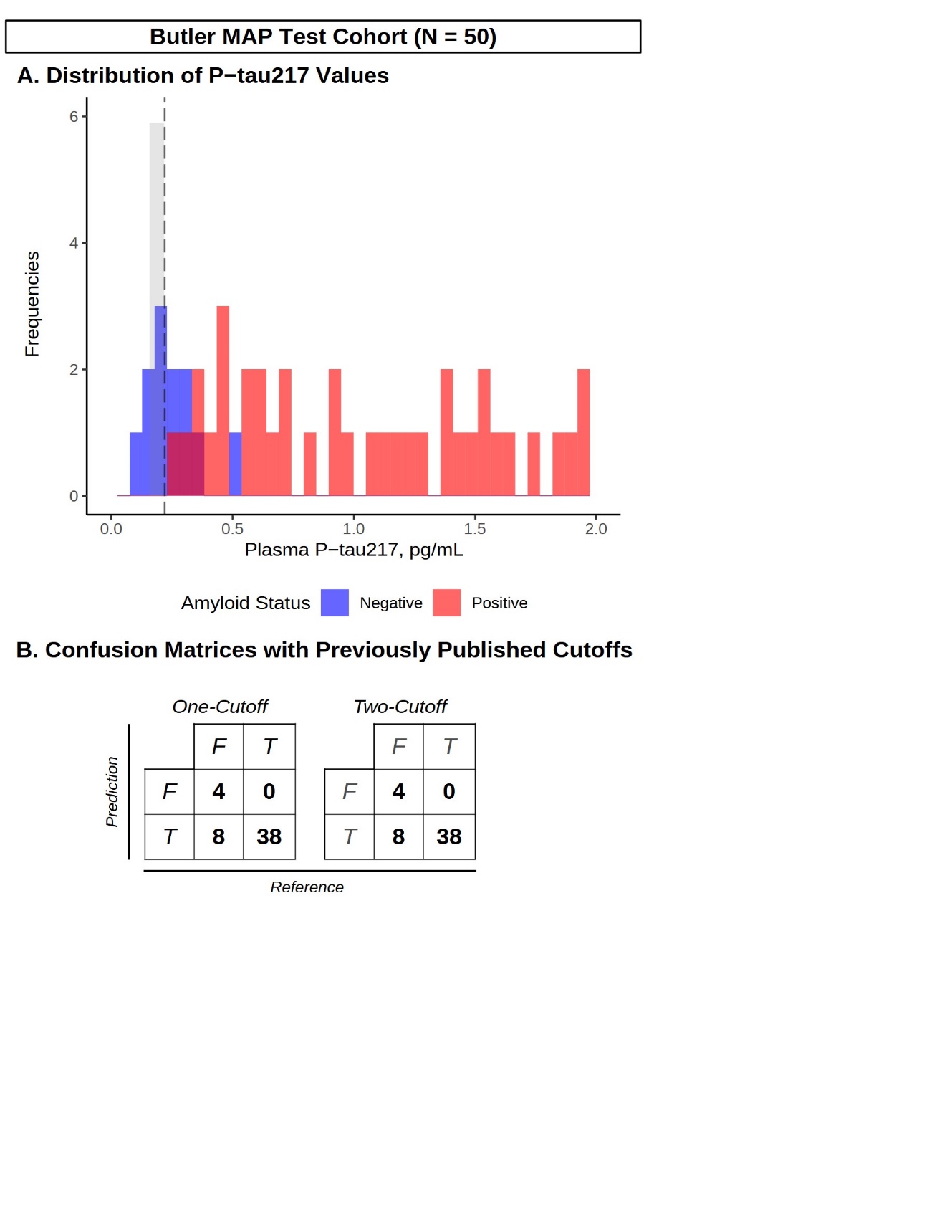


**Supplementary Figure 3.** **Diagnostic performance of previously published cutoffs for DMT eligibility screening. (A)** Histogram depicting the distribution of P-tau217 values in the Test Cohort, with Aβ positive (red) and amyloid negative (blue) participants, as well as Youden’s optimal cutoff (black dashed line) and the intermediate region (shaded gray). **(B)** Confusion matrices for the one- and two- cutoff strategies in the Test Cohort using previously published cutoffs (one-cutoff: 0.22 pg/mL, two-cutoff: 0.159 pg/mL, 0.219 pg/mL) as described from Mattsson-Carlgren *et al.* (2024) compared the reference standard (Aβ-PET/CSF). *N = 50.*

**Reference:**

Mattsson-Carlgren N, Collij LE, Stomrud E, Pichet Binette A, Ossenkoppele R, Smith R, et al. Plasma Biomarker Strategy for Selecting Patients With Alzheimer Disease for Antiamyloid Immunotherapies. JAMA Neurol. 2024 Jan 1;81(1):69–78.
