# Supplementary material for: Clinical application of plasma P-tau217 to assess eligibility for amyloid-lowering immunotherapy in memory clinic patients with early Alzheimer’s disease
Source: Alzheimers Res Ther. 2024 Jul 6;16:154. doi: 10.1186/s13195-024-01521-9 (PMC11227160; doi:10.1186/s13195-024-01521-9)
Supplement: Supplementary file 10 — Additional file 10: Supplementary Fig. 4. DMT eligibility flow charts using BioFINDER-2 cutoffs. [file 13195_2024_1521_MOESM10_ESM.docx]

**(Additional File 10)**


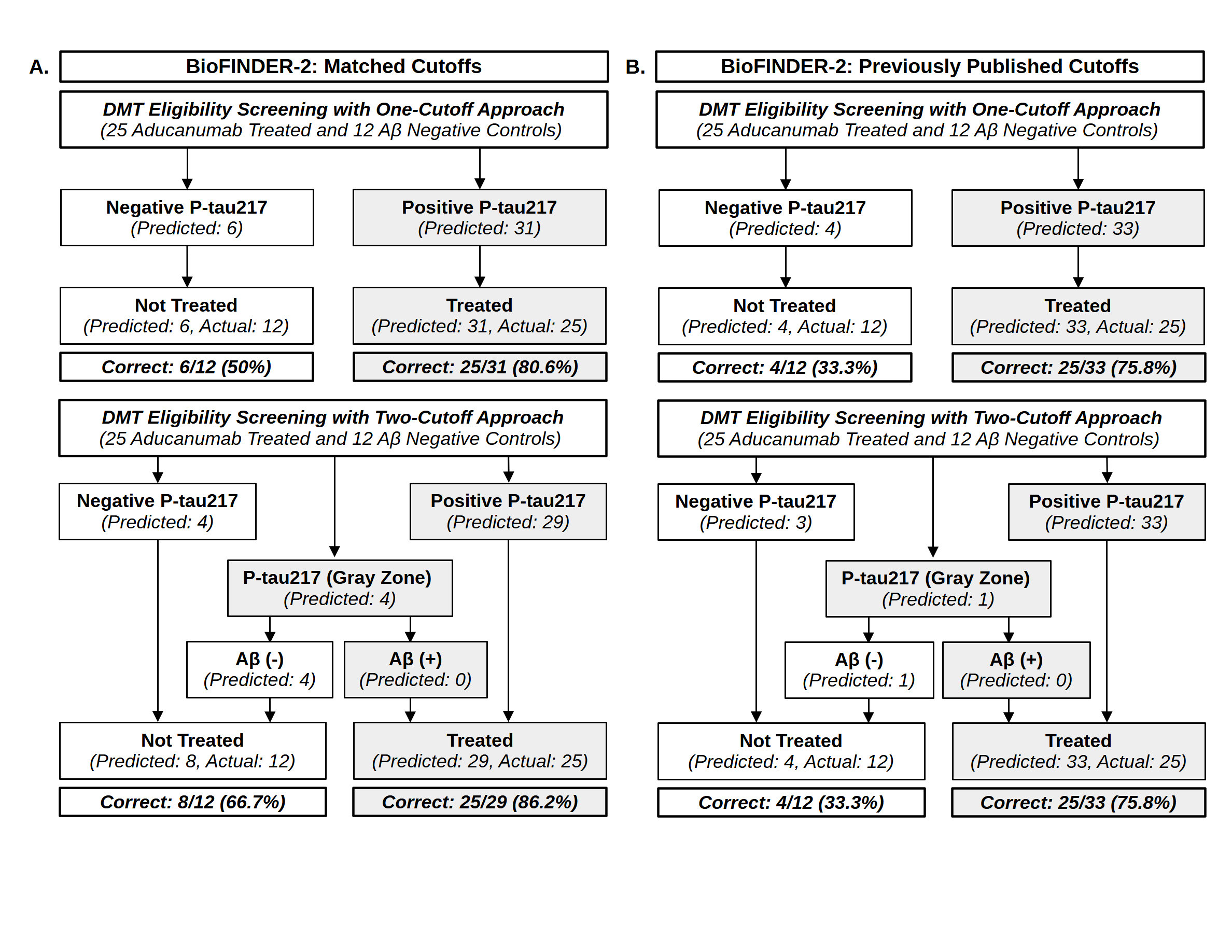


**Supplementary Figure 4. DMT eligibility flow charts using BioFINDER-2 cutoffs.** Analysis of predicted versus actual treatment decisions had P-tau217 been used to determine Aβ positivity in Butler MAP participants treated with aducanumab (n = 25), alongside Aβ negative controls (n = 12). **(A)** One- and two-cutoff predictions using cutoffs from the matched BioFINDER-2 cohort. **(B)** One- and two-cutoff predictions using previously published cutoffs from Mattsson-Carlgren *et al.* (2024). %Correct is calculated by comparing model predictions to the clinical decision to treat with aducanumab based on Aβ-PET/CSF testing. *N = 37.*

Mattsson-Carlgren N, Collij LE, Stomrud E, Pichet Binette A, Ossenkoppele R, Smith R, et al. Plasma Biomarker Strategy for Selecting Patients With Alzheimer Disease for Antiamyloid Immunotherapies. JAMA Neurol. 2024 Jan 1;81(1):69–78.
